# Results of Endovascular Treatment of Ruptured Intracranial Aneurysms and Recanalization Rate After a Five-Year Follow-Up Period

**DOI:** 10.3390/jcm15155756

**Published:** 2026-07-23

**Authors:** Snežana M. Lukić, Vojin S. Kovačević, Dragić Banković, Nemanja Z. Petrović, Nemanja Jovanović, Nenad J. Zornić, Marko Petrović, Stevan Erić, Nikola Mirković, Jelena Nešić, Marina Miletić Kovačević

**Affiliations:** 1 Department of Radiology, Faculty of Medical Sciences, University of Kragujevac, st. Svetozara Markovića 69, 34000 Kragujevac, Serbia; snezanamlukic@gmail.com; 2 Department of Surgery, Faculty of Medical Sciences, University of Kragujevac, st. Svetozara Markovića 69, 34000 Kragujevac, Serbia; nenadzornic@gmail.com (N.J.Z.); markopetrovickg@yahoo.com (M.P.); stevan_kg@yahoo.com (S.E.); drnikolamirkovic@gmail.com (N.M.); 3 Clinic for Neurosurgery, University Clinical Centre of Kragujevac, st. Zmaj Jovina 30, 34000 Kragujevac, Serbia; jovanovich.nemanja@gmail.com; 4 Department of Mathematics and Informatics, Faculty of Science, University of Kragujevac, st. Svetozara Markovića 69, 34000 Kragujevac, Serbia; bankovicdragic@gmail.com; 5 Department of Pharmacology and Toxicology, Faculty of Medical Sciences, University of Kragujevac, st. Svetozara Markovića 69, 34000 Kragujevac, Serbia; dr.nemanja.z.petrovic@gmail.com; 6 Center for Anesthesiology, University Clinical Centre of Kragujevac, st. Zmaj Jovina 30, 34000 Kragujevac, Serbia; 7 Center for Vascular Surgery, University Clinical Centre of Kragujevac, st. Zmaj Jovina 30, 34000 Kragujevac, Serbia; 8 Department of Internal Medicine, Faculty of Medical Sciences, University of Kragujevac, st. Svetozara Markovića 69, 34000 Kragujevac, Serbia; jelenanesic1981@gmail.com; 9 Department of Histology and Embriology, Faculty of Medical Sciences, University of Kragujevac, st. Svetozara Markovića 69, 34000 Kragujevac, Serbia; marina84kv@gmail.com

**Keywords:** ruptured intracranial aneurysms, endovascular embolization, treatment outcome, recanalization

## Abstract

**Background/Objectives:** Endovascular embolization of ruptured intracranial arterial aneurysms has become the first-choice treatment due to its minimal invasiveness, rapid patient recovery, and lower complication rate. The aim of this study was to analyze putative risk factors of unfavorable outcomes of ruptured intracranial aneurysms after coil embolization and the recanalization rate. **Methods:** This case series included adult patients with ruptured intracranial aneurysms who underwent endovascular embolization during the study period of 2007–2023. In total, 1080 patients with ruptured intracranial aneurysms treated using endovascular coiling participated in this study. **Results:** During the first year after the initial intervention, repeated embolization was performed for 50 patients (5.5%). Treatment outcome was assessed using the modified Rankin Scale, and neuroradiological follow-up was conducted during a five-year period on 672 patients. During the period from one to five years, re-embolization was performed in 11 (1.5%) cases, of which only three occurred during the last 3 years of follow-up. **Conclusions:** Endovascular embolization is a safe method for the treatment of ruptured intracranial aneurysms, with a low complication rate and good clinical outcome. Higher risk for repeated aneurysm embolization is associated with male sex, alcohol consumption, smoking, hyperlipidemia, aneurysm size, diabetes, aneurysm location, and obliteration rate. This study showed that aneurysms in which recanalization is not observed within 2 years after coil embolization remain stable during a follow-up period of 5 years, except in the case of large aneurysms and low obliteration rates.

## 1. Introduction

An intracranial aneurysm is a disease caused by local weakness of the arterial wall. If an aneurysm ruptures, there are grave consequences such as subarachnoid hemorrhage (SAH) followed by cerebral vasospasm and cerebral herniation, accompanied by high mortality and disability rates among survivors [1]. Subarachnoid hemorrhage secondary to ruptured intracranial aneurysms is a life-threatening condition with an annual incidence rate of 6–7 per 100,000 in the majority of countries [2]. Endogenous factors such as elevated blood pressure, atherosclerosis, and the special anatomy of the circle of Willis or other brain arteries, especially at their bifurcations, are associated with accelerated growth and increased risk of rupture of aneurysms. Smokers are at increased risk of SAH and cerebral infarction after rupture of an aneurysm [3]. Smoking also requires more intense patient care during endovascular treatment and adversely affects the long-term recovery rate [4].

In patients who present with SAH, high values of Hunt and Hess Grade (HHG) and intraventricular hemorrhage (IVH) were associated with worse outcomes. Many studies have found that primary risk factors for unfavorable outcomes are advanced age, female gender, and smoking, while hypertension plays a higher role in younger patients but does not have much impact on elderly patients. In the literature, there is no evidence of a relation between the rupture risk and older age [5].

Endovascular embolization has become the first choice for the treatment of intracranial aneurysms due to its minimal invasiveness, rapid patient recovery, and low complication rate [6,7,8]. Since the introduction of endovascular embolization in 1991, direct coiling has become an established technique for the treatment of patients with both ruptured and unruptured intracranial aneurysms [9,10]. The angiographic results of coil occlusion can be classified as complete, near-complete, or incomplete using a modified Raymond classification scale (2). Occlusion is “near complete” or “subtotal” when the sac is occluded, but a neck remnant remains. “Incomplete occlusion” can be defined as an aneurysm having loose packing and partial opacification of the aneurysm sac. The distinction between small recurrences (a small change in packing) and full recanalization is determined by the interventional radiologist who performs the procedure. Recanalization, as a consequence of coil compaction due to high arterial blood flow or aneurysm growth, was considered to require a second treatment because of the high risk of rupture [11].

However, only a few studies have reported the short- or midterm results of endovascular coiling, and long-term outcomes have not been investigated. In approximately 20% of patients, the coiled aneurysms reopened during the follow-up [9]. The aim of our study was to identify factors associated with the unfavorable outcome of ruptured intracranial aneurysms after coil embolization and the need for re-embolization in the next few years. Also highlighted as a particular problem is the appropriate length of the follow-up period for patients after endovascular embolization and which risk factors necessitate neuroradiological follow-up over a longer period of time.

## 2. Materials and Methods

This study included a series of adult patients who underwent endovascular embolization (EE) of a ruptured intracranial aneurysm during the study period 2007–2023. In the period from 2007 to 2019, a team of neurointerventional radiologists performed endovascular embolization (EE) at two large medical centers: the University Center with a well-developed neurosurgical department and a general hospital with a neurosurgical department. In the 2019–2020 period, the same team performed EEs at different clinical centers, also with the support of the neurosurgical department, and in the period of 2020–2023 at the main regional Special Hospital for Cerebrovascular Diseases. The results are presented with a neuroradiological follow-up period of 5 years after endovascular embolization. Following the decision of a medical consilium (neurologist, neurosurgeon, and two interventional radiologists) and with the written consent of the family members, the embolization was performed under general anesthesia by the same team of interventional radiologists at the above-mentioned four facilities. Ethical approval was not required for reporting individual cases or case series; the patients or their families signed consent for using their anonymized data for research purposes at admission to the hospital.

The diagnosis of ruptured intracranial aneurysm was made using computerized tomography (CT) angiography, and the endovascular embolization of the aneurysms was performed by insertion of detachable coils, stents, or flow-diverting stents using a DSA system (Axiom Artis, Siemens, Forchheim, Germany, or Alura Philips, Best, the Netherlands). All patients were treated under general anesthesia. Many different coils and stents were used, and selection was conducted according to their availability, the pathological substrate, and preferences of the interventional radiologists (Table 1). The immediate post-coiling digital subtraction angiography (DSA) images served as a reference for follow-up imaging. The initial results of endovascular coiling based on DSA were classified into three categories: complete obliteration (class 1), residual neck (class 2), and residual aneurysm (class 3).

The femoral approach was used, and after angiography, a guiding catheter was advanced to the parent artery, followed by systemic heparinization with 3000–5000 units of unfractionated heparin. During the procedure of endovascular embolization, distal embolization from a thrombus originating from the aneurysm was verified in 91 (8.5%) patients; to these patients, tirofiban was administered intravenously (0.1 μg/kg/min for 24 h). In patients who have had a stent implanted due to a wide entrance to the aneurysm, after verification that they were not resistant to clopidogrel, a dual antiplatelet therapy was administered (clopidogrel 75 mg and aspirin 100 mg, orally) for 6 months, followed by monotherapy with aspirin for the next 6 months. Patients who were resistant to clopidogrel were treated with ticagrelor (Figure 1, Figure 2 and Figure 3). Once the EE was completed, nimodipine was administered to prevent a spasm of intracranial arteries (60 mg/24 h in the first week after SAH, 40 mg/24 h in the second week, and 20 mg/24 h in the third week).

The primary outcome after the treatment in this study was evaluated with the modified Rankin Scale (mRS) score at discharge and after 6 months, when the second control MR angiography was usually performed [12].

### Follow-Up

The CE-MRAs were performed on a superconducting 1.5T MR imaging system (Avanto Siemens, Germany) with a standard head coil using gadobutrol (Gadovist, Bayer Schering Pharma, Berlin, Germany) or gadodiamide (Omniscan, Nycomed, Amersham, Oslo, Norway).

The first CE-MRA was performed 3 months after aneurysm coiling. The next follow-up CE-MRAs were performed after 1 year, 2 years, and 5 years. In cases in which the first CE-MRA suggested concern for aneurysm recanalization, we shortened the interval to the subsequent CE-MRA. The CE-MRA images were axial source images of the brain and MIP reconstructions of the appropriate artery associated with the coiled aneurysm. The DSA images provided were anteroposterior, lateral, and rotational projections obtained at the end of coiling. In the case of recanalization of the aneurysm, the procedure of EE was repeated.

An analysis of the clinical outcome of treatment was conducted only in patients who survived and were not lost during the five-year follow-up period. We consider the clinical outcome to be positive if there are no neurological signs and negative if there is mild to severe disability.

Statistics: The study data were first extracted from the patient files and then tabulated. After the tabulated data were checked and corrected by an independent investigator for errors, descriptive statistical processing was performed. Categorical variables were described by rates and percentages. The significance of differences among the study groups was tested by the chi-square or Fisher’s exact tests if assumptions of the former were not met. One-way analysis of variance was used to test for differences in age among the groups according to the location of the ruptured aneurysm. The occurrence of neurological signs and re-embolization over time was examined using univariate and multivariate Cox regression analyses. The log-rank test was used to investigate the difference in the necessity to perform re-embolization, followed over time, between groups. Kaplan–Meier curves are presented as well. Differences were considered significant at *p* < 0.05. All calculations were made using PASW Statistics for Windows, Version 18.0. Chicago: SPSS Inc (SPSS Inc., Chicago, IL, USA).

## 3. Results

In total, 1080 patients with ruptured intracranial aneurysms treated by EE were included in this study. The median age was 58 years (19–81); there were 670 females (62.0%) and 410 males (38.0%). The risk factors for rupture of an intracranial aneurysm were distributed in our case series as follows: smoking in 858 (79.4%) patients, hereditary burden in 420 (38.8%) patients, hypertension in 892 (82.6%) patients, and hypercholesterolemia in 562 (52.0%) patients. According to heterogeneously obtained data, genetic predisposition was present in 420 (38.8%) patients.

The majority of ruptured aneurysms were located on the internal carotid artery—327 cases (30.3%). Regular-shaped aneurysms were present in 672 (62.2%) patients. In 672 (62.2%) patients, endovascular embolization was performed only with coils, and in 408 (37.8%), it was performed with the use of stents, flow diverter stents, or coiling with balloon assistance. One hundred eighty-six (168–17.2%) patients had more than one aneurysm, and the highest number of diagnosed aneurysms was seven in one patient. Among the patients with multiple aneurysms in the first act, EEs were performed on ruptured aneurysms, and after good clinical recovery, the remaining aneurysms were treated as non-ruptured. Main characteristics of the study patients are shown in Table 2.

There were 707 (65.5%) patients with clinical grade HH I and II after aneurysm rupture; 254 (23.5%) presented with HH grade III; and 119 (11%) with HH grades IV and V. In the first 72 h after aneurysm rupture, EE was performed in 702 (65%) patients and after more than 10 days in 130 (12%) patients. Late embolization after partial recovery of the patient was performed in patients who were in poor clinical grade, presented with spasms, or had concomitant diseases. Considering the size of the aneurysm, those smaller than 10 mm were found in 477 (44%) patients and those larger than 25 mm in 61 (6%) patients. Complete class 1 embolization was performed in 895 patients (82.8%), and the presence of residual aneurysms of class 3 was found in 57 patients (5.3%). After the first embolization, significant technical complications, aneurysm re-rupture, or migration of coils or stents occurred in 21 (1.9%) patients; spasm of arteries was noted in 282 (26.1%), and distal microembolization in 211 (19.5%) patients (Table 3).

The size of the ruptured aneurysms was correlated with the HH grade. Ruptured aneurysms above 10 mm in diameter caused more severe subarachnoid hemorrhage, with worse HH grade. The patients with ruptured aneurysms below 10 mm in diameter had HH grade I or II more frequently (χ^2^ = 57,744; df = 6; *p* ˂ 0.01).

Considering the relation between HH grade and location of the ruptured aneurysm, it was noted that HH grades I-III were more prevalent in patients with ruptured aneurysms of the internal carotid artery and anterior communicating artery than in patients with ruptured aneurysms of other arteries (χ2 = 31.311; df = 6; *p* < 0.01). The HH grades IV and V were more prevalent in patients with ruptured aneurysms of the middle cerebral artery and the basilar artery.

The ruptured aneurysms that had to be re-embolized with coils due to recanalization were most often located on the middle cerebral artery (41 patients) with an average size of 14 mm in diameter and on the internal carotid artery (45 patients) with an average size of 13 mm in diameter. The relatively high rate of re-embolization of aneurysms in these two arteries could be explained by the rapid blood flow and the compaction of the coils.

Patients with rupture of the middle cerebral artery were the youngest within our case series, and those with aneurysms on the posterior circulation were the oldest. The differences in age among the groups according to the location of the ruptured aneurysm were significant (F = 2.611; df = 3; *p* = 0.049).

In our study, clinically significant vasospasm as a complication of spontaneous SAH occurred in 282 (26.1%) patients during the critical period of 4–15 days after the rupture, despite therapy with nimodipine, maintained hypervolemia, and hypertension after the EE. Microembolization of distal intracranial blood vessels, despite the use of dual antiplatelet therapy, was observed in 211 (19.5%) patients on control MRA. Perifocal edema around the embolized aneurysm was present in 322 (29.8%) patients on control MRA during the first three months after embolization.From the initial cohort of 1080 patients, 672 were followed for 5 years in terms of clinical and neuroradiological evaluation, 256 were followed for less than 5 years, and 152 patients were lost to follow-up. None of the patients from our series experienced rebleeding during the follow-up period. Forty patients (3.7%) died in the first 6 months after the EE, and another 46 (4.3%) died between 6 months and 5 years after the EE. Data on patient death were obtained from the patients’ families. The majority of patients who died had major complications after rupture of an aneurysm; most of them were over 65 years old and had multiple comorbidities. Most of the 152 patients who were lost to follow-up were in HH1 grade at admission.

In the first 6 months, re-embolization was performed in 25 (2.4%) patients, while the majority of patients, 50 (5.5%), who underwent re-embolization were treated in the period from 6 months to one year. During the period from one to five years, re-embolization was performed in 11 (1.5%) patients. Looking at the total number of patients (86) who underwent re-embolization, 68 were primarily in the HH3 group, and 18 patients migrated from the HH2 to the HH3 group.

The log-rank test showed that the difference in the necessity to perform re-embolization, followed over time, between the locations of aneurysms was statistically significant (*p* = 0.020). We classified the groups as Location 1 (ACM, ACA, and AcomA), Location 2 (ACI), and Location 3 (posterior circulation). The difference was significant only between Location 1 and Location 2 (*p* = 0.011). Figure 4A shows the Kaplan–Meier curve.

Also, the log-rank test showed that the differences in the need to perform re-embolization, followed over time, between classes of the aneurysm obliteration rates (Class 1, Class 2, and Class 3) are statistically significant (*p* < 0.0005) (Figure 4B). The difference is significant only between Class 1 and Class 2 (*p* < 0.0005). Aneurysm size was also statistically significant (*p* < 0.0005) when considering the need for re-embolization (Size 1 < 10 mm, Size 2 < 15 mm, and Size 3 < 25 mm). The difference was significant between the Size 1 and Size 2 (*p* < 0.0005) groups as well as between Size 1 and Size 3 (*p* < 0.0005), while between Size 2 and Size 3 the difference was not statistically significant (*p* = 0.420) (Figure 4C).

According to risk factor analysis, univariate Cox regression showed that the likelihood of repeated aneurysm embolization, monitored over time, depends on age, sex (85% higher risk in males), alcohol consumption, smoking, hyperlipidemia, aneurysm size (mm), diabetes, aneurysm obliteration rate, and aneurysm location. Multivariate Cox regression showed that the need for re-embolization depends only on aneurysm size (mm) (Table 4).

Three months after EE, which was the first time point after hospital discharge when clinical outcome was evaluated, we found that 62.7% of the patients had a neurologically favorable outcome with an mRS score of 0 or 1; 33.0% of patients had minimal to moderate disability (mRS 2 to 3); and 3.1% had severe disability, which corresponds to mRS grades 4 and 5. At the 5-year follow-up, 91.9% of patients had a good clinical outcome (Table 5).

Univariate Cox regression showed that the negative outcome, followed over time, depends on age, drinking alcohol, smoking, hypertension, hyperlipidemia, positive family history, aneurysm size (mm), diabetes, aneurysm embolization rate, and aneurysm location.

Multivariate Cox regression showed that the negative outcome depends on aneurysm size (mm), diabetes, and the class of aneurysm obliteration rate (Table 6).

## 4. Discussion

Endovascular embolization is nowadays accepted as a complementary method to microsurgery in the treatment of cerebral aneurysms. In certain cases, such as posterior circulation aneurysms and elderly patients with significant comorbidities, it has become the method of first choice [1]. The two most relevant studies, the International Subarachnoid Aneurysm Trial (ISAT) and the Barrow Ruptured Aneurysm Trial (BRAT), reached remarkably uniform conclusions: endovascular coiling provides superior short-term clinical outcomes, but surgical clipping offers vastly superior long-term radiological durability [8,13].

Recanalization is one of the major concerns of endovascular coil embolization for cerebral aneurysms. Our study showed that recanalization and re-treatment rates of embolized aneurysms were 7.9% in the first year and 9.4% in the five years, respectively; this is consistent with previous reports (recanalization rate of 4.7–33.6% and re-treatment rate of 4.7–17.4%) [14,15,16,17,18,19,20,21]. Previous studies reported that risk factors for recanalization were a ruptured aneurysm [16], a wide neck [15], larger-sized aneurysms [22,23,24], incomplete occlusion [21], a low-volume embolization rate [23], and a posterior circulation aneurysm [25]. In our study, a larger aneurysm size was significantly correlated with recanalization, i.e., the risk of re-embolization, according to univariate and multivariate analyses. Incomplete occlusion at the initial procedure was not associated with recanalization in the present study. The reason for this result may be the initially small percentage of Class 3 aneurysms (5.3%). However, a significant difference in recanalization between Class 1 and Class 2 aneurysms was noted. A previous report showed that coils in the acute phase facilitated coil-thrombus formation in the aneurysm, which prevented inflow of blood [26]. On the other hand, a coil-thrombus complex might have resulted in an increase in the volume-packing ratio and could be a reason for the existence of tiny spaces between coils and future recanalization.

In our study, the majority of ruptured aneurysms were in the anterior circulation (78.9%), and accordingly, the prevalence of recanalized aneurysms was higher in the anterior circulation, in contrast to the results of other studies. The reason for this result is the small number of patients with posterior circulation aneurysms who were followed throughout the study. The recanalization rates in general of posterior circulation aneurysms and basilar artery aneurysms are higher than those of aneurysms in other locations [21]. Eleven of the embolized aneurysms out of a total of 86 recanalized aneurysms were re-embolized in a period of 2–5 years after the initial embolization, with just three aneurysms in the last fifth year of follow-up. Therefore, if recanalization is detected within 2 years, we should continue follow-up with imaging in the next few years. Significant risk factors for late changes in coil packing are uncertain, but two of these three aneurysms in the fifth year after coiling were large (>15 mm). We suggest that early recanalized aneurysms should be followed up for >5 years, despite the extent of recanalization, especially in the case of large aneurysms and in the case of Class 2 and 3 obliteration rates.

In our study, aneurysms that were stable for 2 years after the procedure (95.6%) were not recanalized. Raymond et al. [16] previously suggested that all patients should be followed up with non-invasive imaging studies for at least 3 years because 96.9% of all recanalization cases were discovered by 3 years in their study. Furthermore, Holmin et al. [27] showed that stable angiographic findings during a 1-year interval predicted a low risk of recanalization. These findings are compatible with our results. If no recanalization is observed within 2 years after initial endovascular embolization, further annual follow-up imaging studies in relation to treated aneurysms might not be necessary. However, in the present study, the development of new (de novo) aneurysms or the enlargement of tiny or nontreated aneurysms coexisting with treated aneurysms was not evaluated. In managing patients with unruptured aneurysms, risk factors for growth and rupture need to be addressed. Therefore, a follow-up examination after coil embolization should be performed individually while considering de novo and nontreated aneurysms. Awareness of a previous history of subarachnoid hemorrhage and risk factors, such as smoking, hypertension, and family history, is considered important for preventing aneurysmal rupture and growth [28].

Of the total 672 patients for whom we could obtain five-year follow-up results, 91.9% were in good clinical status (without neurological deficits) at the end of the five-year monitoring period. Age, drinking alcohol, smoking, hypertension, hyperlipidemia, aneurysm size, and diabetes were risk factors for the occurrence of neurological deficit. Incomplete aneurysm embolization also turned out to be a significant factor in the occurrence of neurological deficit, which can be linked to the initially poorer clinical grade of the patients and the tendency of the medical team to shorten the embolization procedure in these cases.

We used NMR angiography after endovascular embolization as the standard method for the evaluation of aneurysm recanalization. Additionally, we used non-contrast TOF-MRA routinely to evaluate recanalization of aneurysms, although gadolinium-based enhanced MRA might be more accurate than TOF-MRA [29,30]. Van Amerongen et al. [31] reported that the specificity of TOF-MRA was 84%, with a sensitivity of 86% for the discovery of aneurysm recanalization. For contrast-enhanced MRA, the specificity and sensitivity were 89% and 86%, respectively. However, we did not routinely use contrast-enhanced MRA because our group did not have the uniform technical capabilities in all institutions where we performed endovascular procedures, and the main reason was usually the lack of a contrast injector during the first half of the study period. However, contrast-enhanced MRA shows a higher-quality view of stented parent arteries and fewer artifacts compared with TOF [29].

The early and cumulative mortality rate for the 5-year follow-up period (7.9%) in our study was not significantly higher compared with some recent research [32,33].

Our study has some limitations. First, although a wide neck and the volume embolization rate are correlated with recanalization [34], we were not able to evaluate these factors. We could not collect sufficient data on the size of the neck or the volume embolization rate for further analysis because of the retrospective design of this study. Second, the median follow-up span was 5 years, but a relatively large number of patients with embolized aneurysms could not be followed completely, since of the initial number of embolization procedures performed, complete 5-year follow-up was available for only 62.2% of patients. Earlier follow-up cases were usually excluded because those patients stopped coming to control visits. Also, the group of patients with good early clinical recovery did not have long-term neuroimaging surveillance. In our study, one year after embolization, 12.8% of patients were lost to follow-up.

Moreover, it is unclear whether a non-recanalized aneurysm within 2 years of embolization will be safe for decades or a lifetime, as late recanalization is likely to occur in the future. Additional studies on this issue over a longer period of time may be needed.

Regarding the impact on clinical outcome, there is also a lack of very important parameters, such as neurological deficit on admission and during early hospitalization. The most common cause of this is the sedation of the patient during transport to a specialized facility and the next few days after the embolization procedure. One more significant shortcoming of our study is the lack of data on the total number of patients who experienced aneurysm rupture and were not treated with endovascular procedures during the study period. The reason for this is the partial work engagement of our team in an institution where patients were referred by neurosurgeons, and it was previously decided that the patient was not a candidate for open surgery. In institutions where there was a neurosurgical department, all patients with significant intracerebral hemorrhage were generally treated with clipping.

## 5. Conclusions

We can conclude that endovascular embolization is a safe method for the treatment of ruptured intracranial aneurysms, with a low complication rate and good clinical outcome, except in cases where there is significant intracerebral bleeding. Our study showed that the results of the clinical outcome depend on the size and location of the ruptured aneurysm. Higher risk for repeated aneurysm embolization is associated with male sex, alcohol consumption, smoking, hyperlipidemia, aneurysm size, diabetes, aneurysm location, and obliteration rate. This study also showed that aneurysms in which recanalization is not observed within 2 years after coil embolization are stable during a follow-up period of 5 years, except in the case of large aneurysms and low obliteration rates. Our case series may inform clinicians to set the appropriate frequency of follow-up imaging in patients with previously embolized cerebral aneurysms.

## Figures and Tables

**Figure 1:**
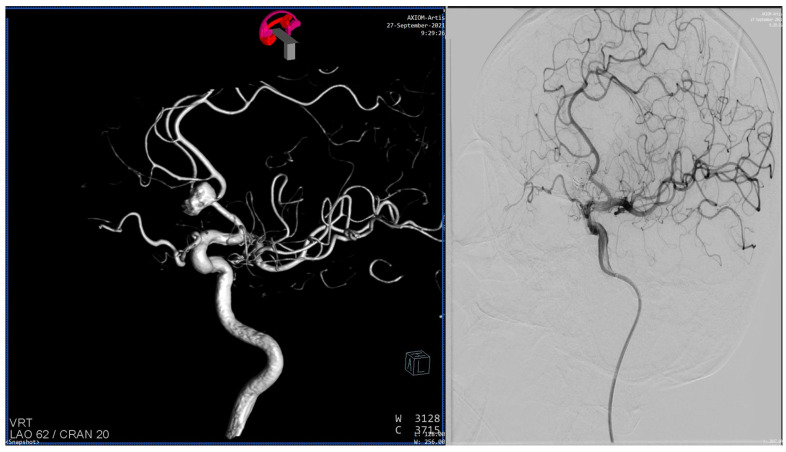
A 67-year-old patient. Ruptured aneurysm of anterior communicating artery, size 9 mm × 8 mm, neck 2 mm wide. There is subarachnoid and intracerebral haemorrhage, clinical status HH4. Endovascular embolization was performed with coils, 2 days after the rupture. After 5 weeks of treatment, clinical status was mRS-2.

**Figure 2:**
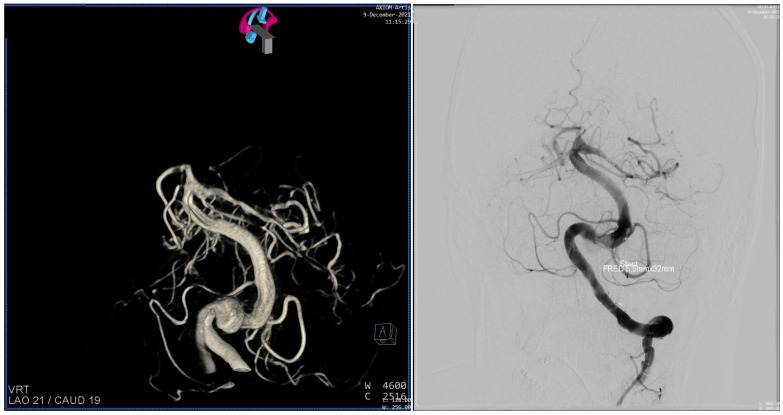
A 61-year-old patient. Ruptured aneurysm of basilar artery, size 9 mm × 8 mm with neck 7 mm wide. SAH, clinical status HH3. The flow divert stent FRED was implanted. Clinical status was mRS-0 on discharge from the hospital.

**Figure 3:**
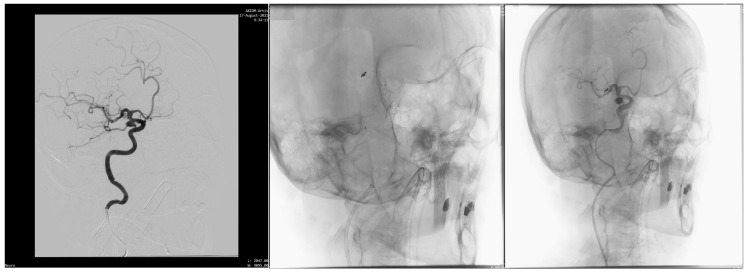
A 70-year-old patient. Ruptured aneurysm of internal carotid artery. Size 7 mm × 5 mm, aneurysm neck 4.5 mm wide. SAH, clinical grade HH3. The Enterprise stent was implanted and coils were placed in the aneurysm through the stent. The clinical status was mRS-1 on discharge from the hospital.

**Figure 4:**
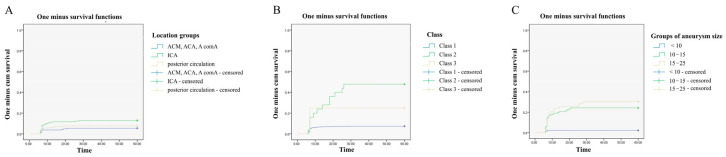
Dependence of the need for reembolization on the aneurysm location (**A**), obliteration rate (**B**) and aneurysm size (**C**).

**Table 1:** List of types of coils and stents used.

Coils	Stents	Flow-Diverter Stents
GDC™ 10 Detachable Coils (Stryker Neurovascular, Fremont, CA, USA)	Neuroform3™ Microdelivery Stent System (Stryker Neurovascular, Fremont, CA, USA)	Silk flow divert stent (Balt Extrusion, Montmorency, France)
Matrix2™ 360 UltraSoft SR Detachable Coils (Boston Scientific Neurovascular, Fremont, CA, USA)	Enterprise stent (Cordis Neurovascular, Fremont, CA, USA)	Papiline flow divert stent (Phenox GmbH, Bochum, Germany)
AXIUM 3D DETACHABLE COILS (Micro Therapeutics, Inc. d/b/a ev3 Neurovascular, Irvine, CA, USA)	Acclino flex stent (Acandis GmbH & Co. KG, Pforzheim, Germany)	Surpass Evolve^®^ flow divert stent (Stryker Neurovascular, Fremont, CA, USA)
Barricade Coil System (Blockade Medical, Irvine, CA, USA)	leo + and leo + baby (Balt Extrusion, Montmorency, France)	Fred TM flow divert stent (MicroVention, Inc., Tustin, CA, USA)
MicroPlex^®^ Coil System Cosmos^®^ 10 Coil (MicroVention, Inc., Tustin, CA, USA)		Derivo flow divert stent (Acandis GmbH & Co. KG, Pforzheim, Germany)
HYDROGEL COILS HydroCoil^®^ Embolic System (MicroVention, Inc., Tustin, CA, USA)		
MICRUSPHERE@ MICROCOIL CERECYTE 10 SYSTEM (Micrus Endovascular Corporation, San Jose, CA, USA)		
Smart coil (Penumbra, Inc., Alameda, CA, USA)		
Target 3D coil (Stryker Neurovascular, Fremont, CA, USA)		
Target nano coil (Stryker Neurovascular, Fremont, CA, USA)		

**Table 2:** Characteristics of the case series (*n* = 1080).

Variable	Value: Rate (%)
**Age**	Median age 58 years, range 19–81 years
**Sex**	Women 670 (62%), men 410 (38%)
**Drinking alcohol**	582 (53.9%)
**Smoking**	858 (79.4%)
**Hypertension**	892 (82.6%)
**Hyperlipidaemia**	562 (52%)
**Positive family history**	420 (38.8%)
**Shape**	
**Regular**	672 (62.2%)
**Irregular**	408 (37.8%)
**Location**	
**Internal carotid artery (ICA)**	327 (30.3%)
**Anterior communicating artery (AComA)**	314 (29%)
**Middle cerebral artery (MCA)**	134 (12.4%)
**Posterior communicating artery (PComA)**	123 (11.36%)
**Anterior cerebral artery (ACA)**	78 (7.22%)
**Basilar artery (BA)**	57 (5.27%)
**Vertebral artery (VA)**	38 (3.52%)
**Posterior cerebral artery PCA)**	9 (0.8%)
**Treatment**	
**Coiling**	672 (62.2%)
**Stent-assisted coiling**	278 (25.7%)
**Balloon-assisted coiling**	12 (1.1%)
**Flow-diverting stent**	118 (10.9%)
**Death**	40 (3.7%)

**Table 3:** The data about the first embolization (*n* = 1080).

Variable	Value (Number (%))
**Neurological status after the aneurysm rupture (HH grade)**	
**Good (HHI-II)**	707 (65.5%)
**Moderate (HH3)**	254 (23.5%)
**Poor (HH4-HH5)**	119 (11%)
**Time interval between an aneurysm rupture and EE**	
**Early (0–3 days)**	702 (65%)
**Intermediate EE (4–10 days)**	248 (23%)
**Late EE (11–30 days)**	97 (9%)
**Delayed (31–60 days)**	33 (3%)
**Aneurysm size (mm)**	
**≤10**	477 (44%)
**10–15**	363 (33%)
**15–25**	179 (27%)
**>25**	61 (6%)
**The outcome of the first EE as visible on DSA:**	
**class 1 (complete obliteration)**	895(82.8%)
**class 2 (residual neck)**	128 (11.8%)
**class 3 (residual aneurysm)**	57 (5.3%)
**Procedural and postprocedural complication**	
**Re-ruptured aneurysm**	7 (0.6%)
**Coil migration**	8 (0.7%)
**Stent migration**	6 (0.5%)
**Spasm**	282 (26.1%)
**Distal microembolisation**	211 (19.5%)
**Perifocal oedema**	322 (29.8%)
**Hydrocephalus**	96 (8.9%)

**Table 4:** Re-embolization and risk factors—results of Cox regression.

	Univariate Cox Egression		Multivariate Cox Regression	
	Risk Ratio (95% CI)	*p*	Risk Ratio (95% CI)	*p*
Age	1.036 (1.002–1.071)	0.040	0.984 (0.947–1.023)	0.411
Male	1.850 (1.047–13.270)	0.034	1.188 (0.635–2.221	0.590
Drinking alcohol	2.181 (1.238–3.840)	0.007	1.080 (0.495–2.357)	0.847
Smoking	4.084 (2.019–8.261)	0.018	1.477 (0.680–3.204)	0.324
Hypertension	2.457 (0.930–5.399)	0.055		
Hyperlipidaemia	2.030 (1.136–3.628)	0.017	1.291 (0.650–2.562)	0.466
**Aneurysm size (mm)**	1.413 (1.309–1.525)	**<0.0005**	1.269 (1.195–1.347)	**<0.0005**
Diabetes	2.278 (1.281–4.044)	0.005	1.370 (0.761–2.467)	0.293
Positive family history	0.479 (0.174–1.320)	0.155		
Obliteration rate (Class2 vs. Class1)	7.011 (3.722–13.209)	<0.0005	0.675 (0.342–1.333)	0.257
ICA vs. {ACM, ACA, AcomA}	2.432 (1.195–4.947)	0.014	ICA vs. {ACM, ACA, AcomA}	0.285

**Table 5:** The results of the follow-up.

Variable	Duration of Follow-Up			
	<3 *Months* *n* = 1040 (96.3%)	≤12 *Months* *n* = 902 (83.5%)	*2 Years* *n* = 721 (66.7%)	*5 Years* *n* = 672 (62.2%)
**Recanalization**				
**Class 1.**	890 (85.6%)	775 (85.9%)	689 (95.6%)	650 (96.7%)
**Class 2.**	110 (10.6%)	75 (8.3%)	25 (3.5%)	18 (2.7%)
**Class 3.**	40 (3.8%)	52 (5.8%)	7 (0.9%)	4 (0.6%)
**Re-embolization**	25 (2.4%)	50 (5.5%)	58 (8.0%)	61 (8.5%)
**Clinical outcome**				
**Good** (without neurological signs, mRS = 0–1)	652 (62.7%)	682 (75.6%)	621 (86.1%)	618 (91.9%)
**Minimal to moderate disabilty** (hemiparesis, episodic headache, mRS = 2–3)	356 (33.0%)	176 (19.5%)	78 (10.8%)	42 (7.3%)
**Severe disability** (hemiplegia, aphasia, **mRS = 4–5**)	32 (3.1%)	42 (4.6%)	32 (4.4%)	12 (1.8%)
**Death cumulatively (mRS-6)**	40 (3.7%)	50 (4.6%)	68 (6.3%)	86 (7.9%)

**Table 6:** Negative outcome and risk factors—results of Cox regression.

	Univariate Cox Egression		Multivariate Cox Regression	
	Risk Ratio (95% CI)	*p*	Risk Ratio (95% CI)	*p*
Age	1.099 (1.064–1.135)	<0.0005	1.003 (0.962–1.046)	0.885
Male	0.907 (0.527–1.561)	0.725		
Drinking alcohol	5.128 (2.748–9.569)	<0.0005	2.174 (0.848–5.578)	0.106
Smoking	4.084 (2.019–8.261)	<0.0005	0.808 (0.287–2.273)	0.686
Hypertension	5.623 (1.755–18.017)	0.004	0.948 (0.242–3.710)	0.939
Hyperlipidaemia	2.030 (1.136–3.628)	0.017	1.966 (0.722–5.349)	0.186
**Aneurysm size (mm)**	1.374 (1.292–1.461)	**<0.0005**	1.163 (1.079–1.253)	**<0.0005**
**Diabetes**	13.382 (6.901–25.952)	**<0.0005**	3.918 (1.829–8.392)	**<0.0005**
Positive family history	0.125 (0.017–0.101)	0.139		
**Embolization rate (** **Class2 or Class3** **)**	39.033 (22.816–66.775)	**<0.0005**	3.971 (1.851–8.521)	**<0.0005**
Location group *		0.672		

* Location group is categorical variable with three categories.

## Data Availability

The datasets generated during and/or analysed during the current study are available from the corresponding author on reasonable request.

## Abbreviations

CE-MRA	Contrast-Enhanced Magnetic Resonance Angiography.
EE	endovascular embolization.
HHG	Hunt & Hess Grade.
ICH	intracerebral haemorrhage.
IVH	intraventricular haemorrhage.
MIP	Maximum Intensity Projection.
NMR	Nuclear Magnetic Resonance.
SAH	subarachnoid hemorrhage.
TOF-MRA	Time-of-Flight Magnetic Resonance Angiography.
